# Sturge-Weber Syndrome: A Report of a Rare Case

**DOI:** 10.7759/cureus.51110

**Published:** 2023-12-26

**Authors:** Osama A Sherwani, Padma C Patra, Syed A Ahmad, Shamimul Hasan

**Affiliations:** 1 Dental Surgery, Uttar Pradesh Provincial Medical Services, Bulandshahr, IND; 2 Dentistry, Faculty of Dental Sciences, Manav Rachna International Institute of Research and Studies, Faridabad, IND; 3 Otolaryngology, Park Hospitals, Gurugram, IND; 4 Oral and Maxillofacial Surgery, Faculty of Dentistry, Jamia Millia Islamia, New Delhi, IND; 5 Oral Medicine and Radiology, Faculty of Dentistry, Jamia Millia Islamia, New Delhi, IND

**Keywords:** tram track calcification, radiographic imaging, sturge-weber syndrome, port wine stains, gingival hypertrophy

## Abstract

Sturge-Weber Syndrome (SWS) is a rare congenital developmental disorder that arises from the abnormal persistence of the embryonic vascular plexus. The syndrome encompasses hamartomatous malformations that can impact the skin, eyes, and nervous system. The broad array of clinical manifestations and potentially life-threatening complications underscores the crucial and imperative need for an accurate diagnosis. An effective treatment strategy for SWS patients involves a multidisciplinary approach. Dental procedures in these individuals pose challenges due to the potential risk for substantial bleeding during both intra- and postoperative phases.

This article aims to document a rare case of Sturge Weber Syndrome in a 21-year-old female patient who presented with seizures, unilateral facial port wine stains, gingival hyperplasia, and intracranial calcifications.

## Introduction

Sturge-Weber Syndrome (SWS), referred to as encephalo-facial angiomatosis or encephalo-trigeminal angiomatosis, is a rare disorder classified as a mesodermal phakomatosis ("mother-spot" disease). Facial port wine vascular nevus, leptomeningeal, and choroidal angiomas, associated with glaucoma, seizures, neurologic deficits, and eventual neuro-developmental delay are the cardinal presenting features [1].

Schirmer provided the initial comprehensive description of SWS in 1860, and Sturge elucidated the cutaneous, ocular, and neurological manifestations in 1879. Furthermore, Weber documented radiological changes observed in these live births [1-3].

The exact incidence of this syndrome is not precisely reported, and it is estimated to manifest in approximately 1 in 20,000-50,000 live births. SWS does not exhibit a gender or racial predilection [1,4].

Oral features encompass unilateral haemangiomatous lesions in the maxillary or mandibular gingiva, tongue, lips, and palatine region. Gingival lesions characteristically manifest as unilateral hypertrophy due to increased vascularity and exhibit profuse bleeding on gentle manipulation [1,2].

Establishing a diagnosis involves a thorough clinical assessment. However, an affirmative diagnosis is typically achieved through imaging techniques like plain X-ray, computed tomography (CT), and magnetic resonance imaging (MRI) scans [5]. Gadolinium-enhanced MRI is the primary imaging technique for assessing neural involvement in SWS [4].

There is no specific treatment for SWS, and effective management necessitates an interdisciplinary approach. The medical intervention involves anticonvulsant therapies for seizures, symptomatic and preventive measures for headaches, glaucoma management to decrease intraocular pressure, and laser treatment for port-wine stains (PWS) [1,6]. Nevertheless, recalcitrant cases of seizures require surgical intervention [5,7].

The significant occurrence of oral manifestations in SWS emphasizes the necessity for an oral health physician's comprehensive understanding of this rare congenital disorder. Regular dental and oral surgical procedures may be complicated by the recognized risk of intra- and postoperative bleeding [1].

## Case presentation

A 21-year-old, mentally subnormal female patient presented at our Outpatient Department with a complaint of bleeding and swelling in the gums. She was the oldest among four siblings, born after a full-term, uncomplicated pregnancy through normal delivery. The patient's medical history indicated the presence of a reddish discolored patch on the left side of the face since birth. Initially, it was small, but it progressively enlarged with overall body growth to its current size and darkened over the years. History also revealed that she experienced convulsive episodes at 1.5 years of age, for which she was taking on-and-off homeopathic medications prescribed by a private practitioner. The specific details of the medical prescription were not available. Initially, the patient had 1-2 convulsive episodes/day. Nevertheless, the frequency of convulsive episodes has progressively diminished over time, and she experienced a convulsive episode four months ago. The family history of any presenting illness in any individual from the maternal or paternal side was non-contributory.

**Figure 1:**
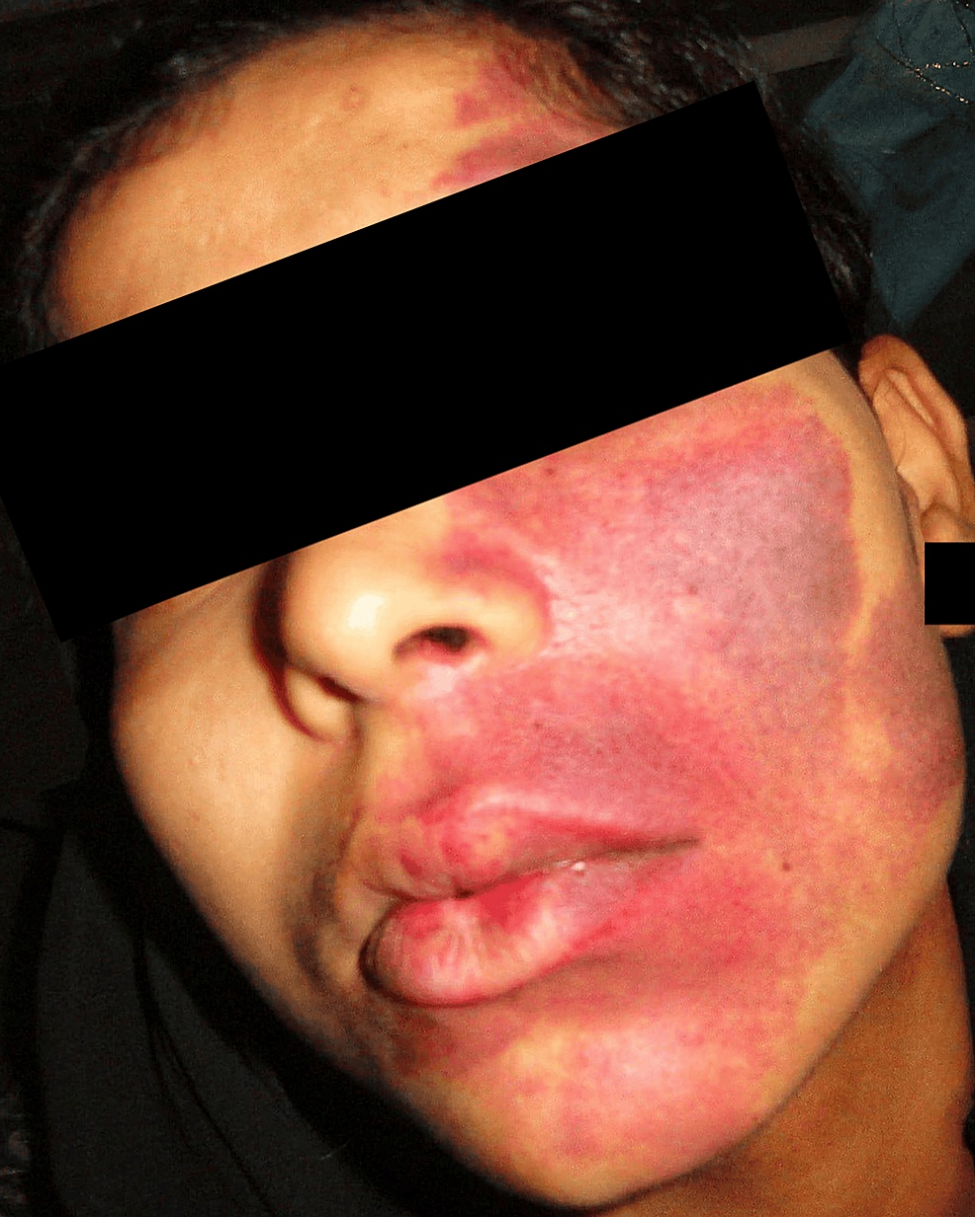
Clinical picture showing a diffuse reddish-purplish patch (port-wine stain) on the left side of the face.

Physical examination revealed a diffuse, flat reddish-purple patch (PWS) on the left side of the face over the areas supplied by the three branches of the trigeminal nerve. It extended from the midline of the forehead, reaching superiorly up to the hairline, inferiorly down to the lower border of the mandible, laterally up to the left ear, and medially crossing the midline in the chin region. The discolored patch exhibited an irregular shape with clearly defined edges. The upper lip appeared swollen, and edematous and exhibited blanching on pressure. On palpation, the patch was flat, smooth, non-tender, and warm to the touch. No visible pulsations were apparent (Figure 1).

**Figure 2:**
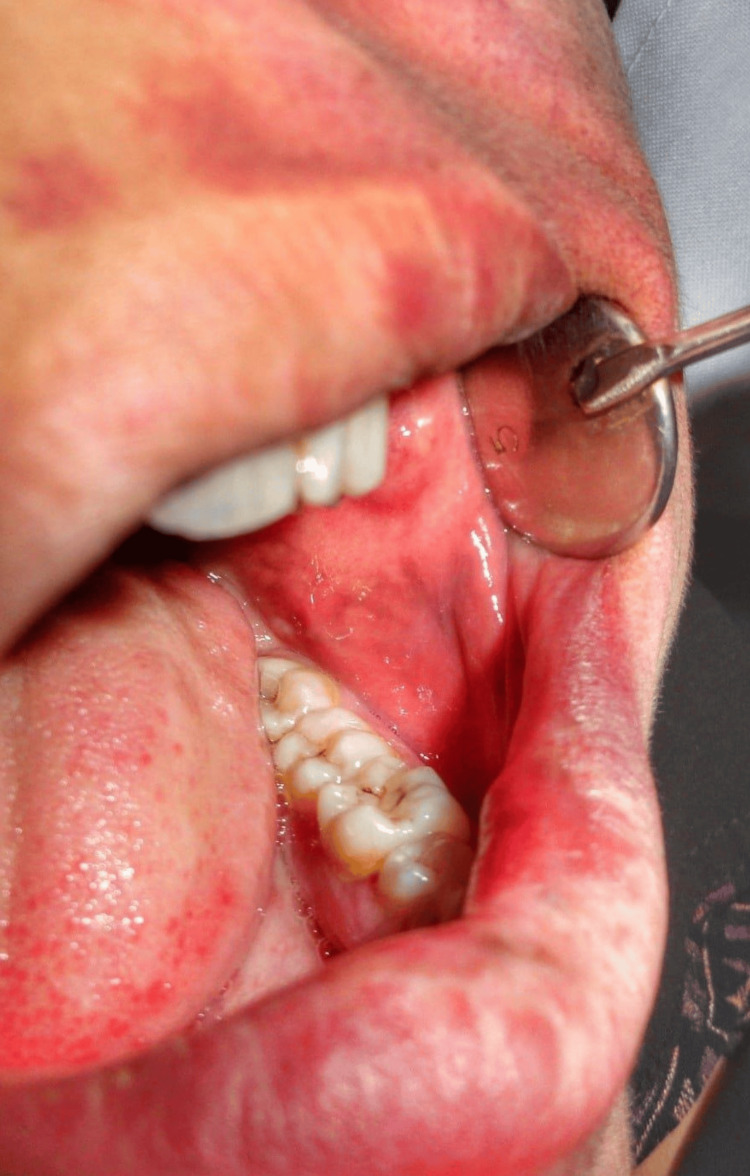
Intraoral picture showing port-wine stain involving the left buccal mucosa and lower labial mucosa.

On intraoral examination, a well-defined extension of the PWS was evident on the left side, involving the left buccal mucosa and lower labial mucosa (Figure 2).

**Figure 3:**
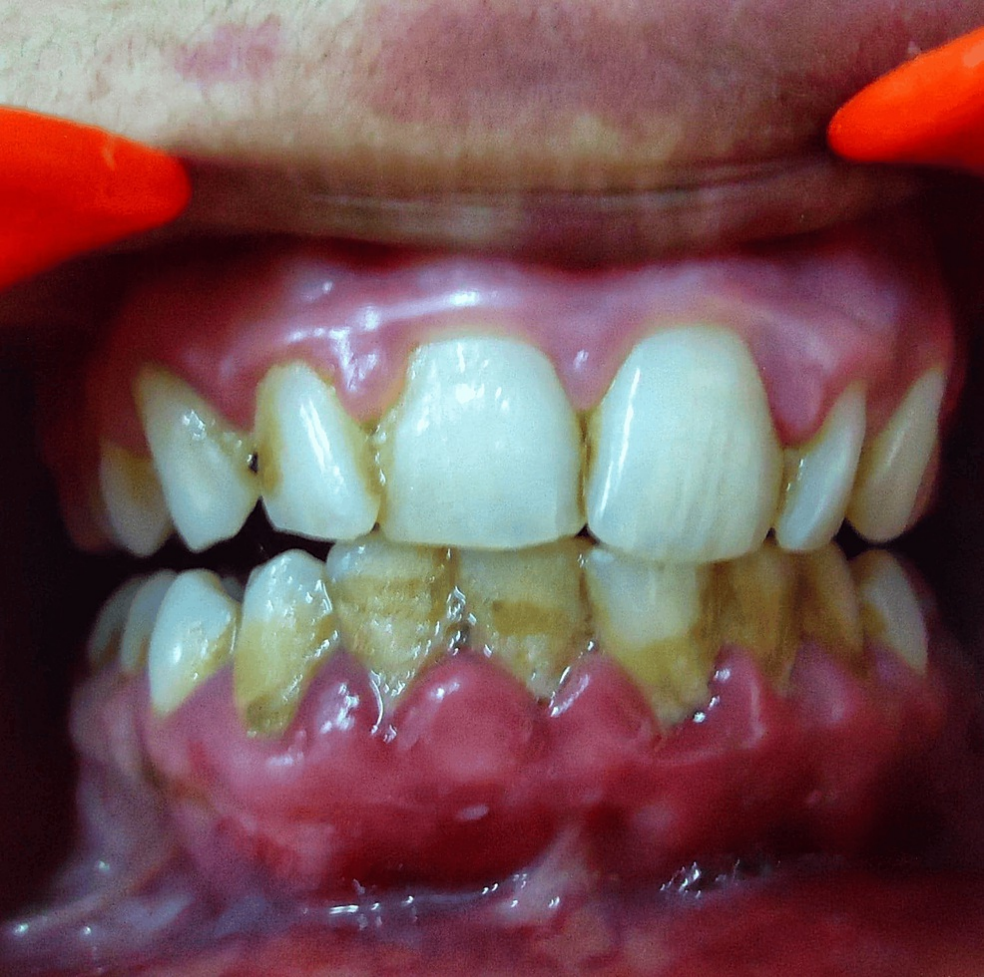
Intraoral picture showing gingival enlargement.

Interdental, marginal, and attached gingival hyperplasia was observed in the maxillary and mandibular anterior region. The hyperplastic gingiva appeared bright red, had a soft consistency, bled with gentle manipulation, and showed blanching under pressure, indicating an angiomatous enlargement. The patient had poor oral hygiene with heavy plaque and calculus (Figure 3).

**Figure 4:**
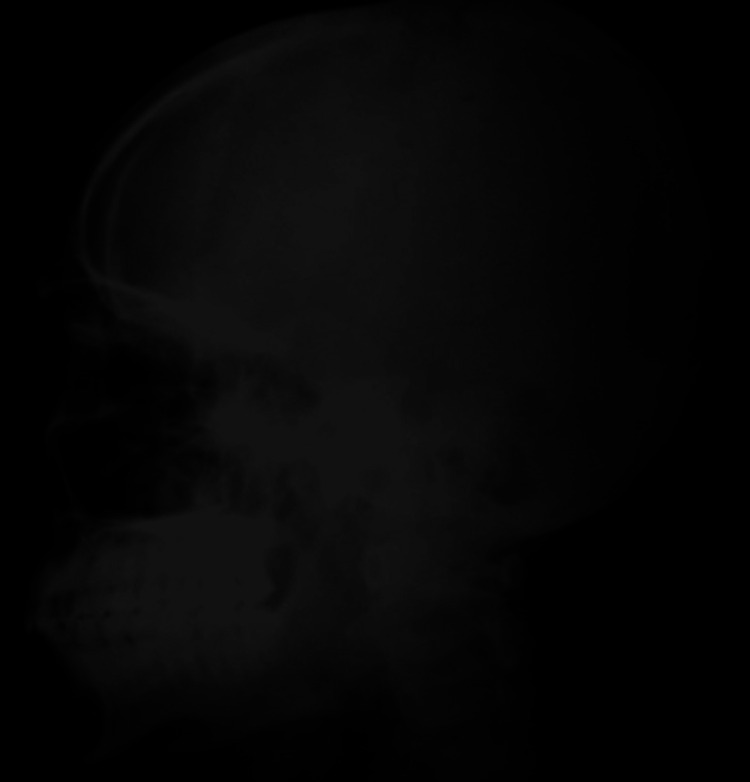
Lateral skull radiograph showing mild eminence of vessels.

The left lateral skull radiograph revealed slight eminence of the vessels with mild interdental bone loss around the teeth (Figure 4).

**Figure 5:**
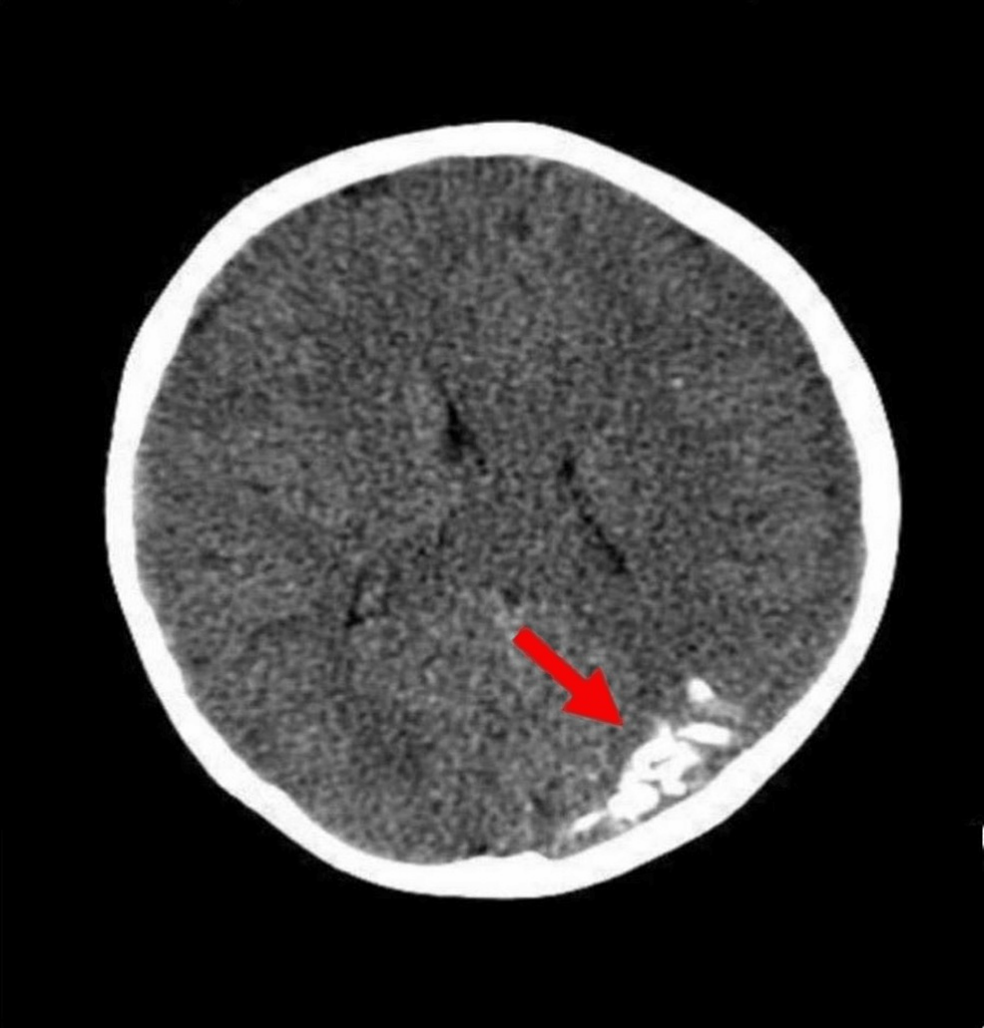
CT scan revealing gyriform calcifications in the left parieto-occipital region. The red arrow depicting the typical intracranial calcifications (tram track appearance).

A CT scan revealed hyperdense lesions arranged as parallel, subcortical gyriform calcifications (tram-track appearances) in the left parieto-occipital region (Figure 5).

A definitive diagnosis of SWS was established based on the history of convulsive episodes, clinical presence of a unilateral facial PWS, angiomatous gingival hypertrophy, and radiographic evidence of intracranial calcifications.

Hematological investigations including complete blood count, bleeding, and clotting time were in the normal range. The treatment procedures were explained to both the patient and their parents, and written informed consent was taken. Medical clearance from the physician was also obtained before the treatment.

The primary therapeutic aim was to ameliorate the patient's periodontal health. This involved implementing a comprehensive plaque control program, including regular deep scaling and root planing, motivating the patient to maintain optimal oral hygiene, the use of 0.2% chlorhexidine mouth rinse after brushing, along with a balanced diet regimen.

**Figure 6:**
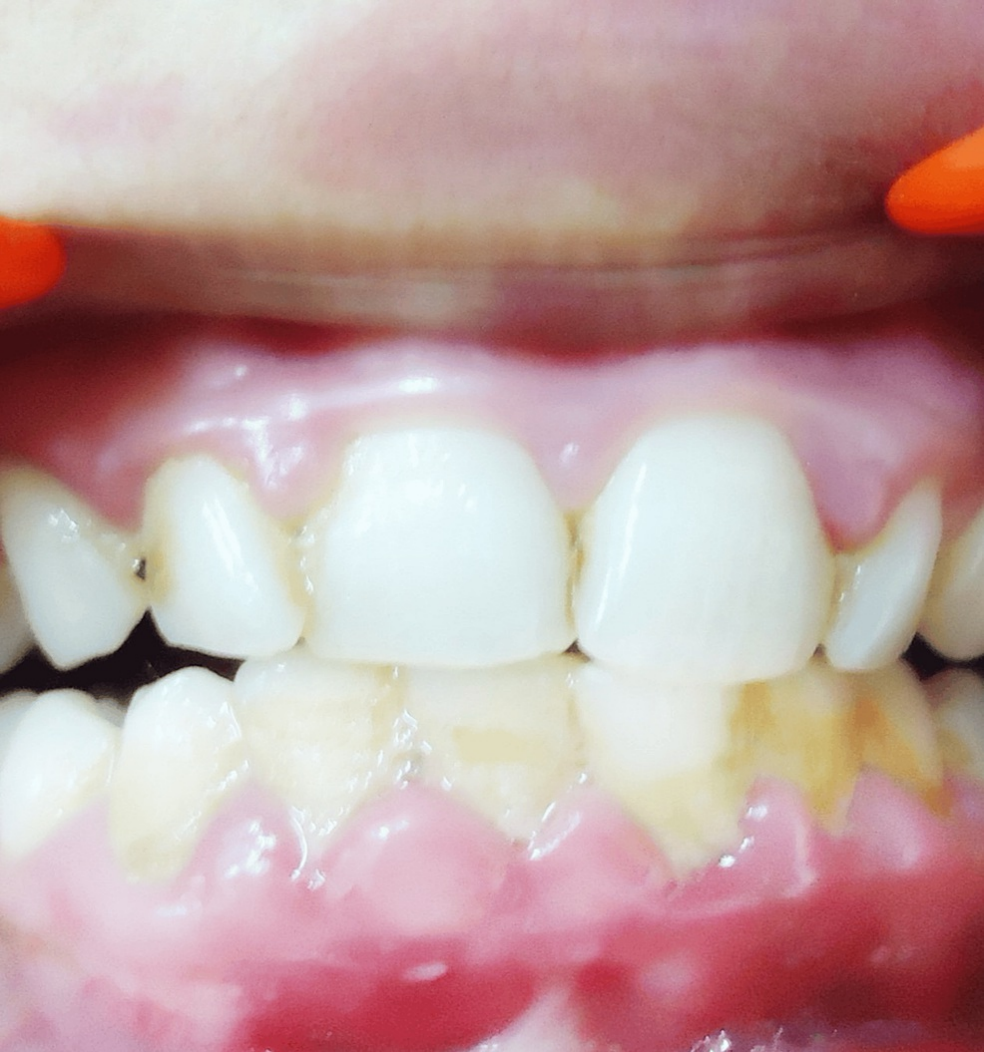
Post-treatment picture

A significant reduction in the degree of gingival enlargement and no bleeding on manipulation was observed after the one-month follow-up (Figure 6).

Regular follow-up and complete plaque control carried out every three months showed a remarkable improvement in the periodontal condition of the patient.

## Discussion

SWS is a congenital, sporadic, and rare neurocutaneous disorder, characterized by the presence of a facial port-wine birthmark (PWB), glaucoma, and leptomeningeal angiomatosis [4]. After neurofibromatosis and tuberous sclerosis, it is recognized as the third most prevalent neurocutaneous syndrome [4,6].

SWS arises due to post-zygotic, somatic gain-of-function variants in the GNAQ gene located on the long arm of chromosome 9. Nevertheless, recent descriptions have emerged concerning GNA11 variants, revealing subtle phenotypic differences [8]. SWS is an embryonic developmental anomaly that arises from the partial regression of a vascular plexus surrounding the cephalic region of the neural tube, intended to develop into facial skin. Normally, this vascular plexus forms during the sixth week of intrauterine life and exhibits regression by the ninth week. The endurant dysfunction of an embryonal vascular system results in angiomas due to residual vascular plexus in the brain and eye [1,3,9]. A “vascular steal phenomenon” may develop around the angioma in the leptomeninges of the brain leading to the pooling of blood and obstruction to venous return creating difficulty in the entry of fresh blood to affected regions of the brain which suffers ischemic insult, undergoes atrophy, and shows calcifications. Neurological dysfunction such as recurrent seizures, status epilepticus, intractable seizures along with recurrent vascular events can propagate this steal further, with an elevation in cortical ischemia, resulting in progressive calcification, gliosis, and atrophy leading to seizures and neurological deterioration [1].

PWS on the forehead and/or scalp, seizures and mental retardation due to leptomeningeal angiomas, and glaucoma constitute the cardinal features of SWS [2]. PWS, or nevus flammeus, are cutaneous capillary malformations present at birth over the forehead and nose. They overlay the distribution of trigeminal nerve branches (V1, V2, and V3) and endure throughout an individual's life [10].

PWS are observed in approximately 87-90% of cases, commonly appearing on the right side. However, in about 33% of cases, these lesions can manifest bilaterally [2]. Sturge-Weber angiomatosis may not be present in all individuals with facial PWS, and the involvement of the region supplied by the ophthalmic division is deemed pathognomonic [3].

The anatomic location of vascular malformation has been linked with the risk of developing leptomeningeal angiomas and glaucoma, with a greater likelihood when the PWS is situated in the frontal region compared to those located in the lower facial region. There is also evidence suggesting that the more extensive the lesion, the more severe the neurological involvement [4,6,11].

Our patient presented with a diffuse reddish-purplish patch (PWS) on the left side of the face in the areas supplied by the trigeminal nerve. The PWS was present since birth and has darkened with age.

Untreated capillary malformations may progress and cause hypertrophy of the soft/bone tissue, or nodule within the affected structures [12]. Oral features observed in SWS are attributed to the hemangiomatous oral lesions [6,13].

Oral vascular lesions may vary from solitary vascular hyperplasia to pronounced angiomatous proliferation or pyogenic granulomas, thus, rendering the mucosa fragile to minimum trauma and minor dental procedures. These lesions exhibit a distinct demarcation in the midline and are located on the same side as the facial lesion [4]. Gum hypertrophy in SWS may be due to the angiomatous growth of the affected gingival tissues, drug-induced (anticonvulsant therapy), or a combination of both factors [14].

In our patient, the gingival hypertrophy could possibly be due to the angiomatous growth superimposed with poor oral hygiene.

Hard tissue involvement consists of bone hypertrophy and tooth malpositioning. In certain cases, macroglossia and maxillary bone hypertrophy may lead to malocclusion and a dysmorphic facial appearance [2,6,13].

Leptomeningeal angiomatosis also depicts a noteworthy clinical manifestation of SWS, potentially causing contralateral hypertrophy, cerebral calcification, epileptic seizures, and cognitive impairment [2]. Generally, leptomeningeal angiomas are detected unilaterally, primarily in the occipital and parietal regions [3]. More than 70% of children affected by SWS experience partial seizures by the age of three, and between 50% and 75% of these children exhibit some degree of developmental delay or mental retardation [6,15,16].

Our patient had convulsive episodes since the age of one and a half years, for which she was taking on-and-off homeopathic medications prescribed by a private medical practitioner.

Ocular involvement can result in glaucoma, choroidal hemangioma, buphthalmos, or hemianopia [3].

Dilated blood vessels in the left eye with areas of congestion were observed in the present case.

SWS is categorized as complete when it involves both facial and central nervous system (CNS) angiomas, and as incomplete when it affects only one area without involving the other [3]. The Roach scale classifies SWS into three types: Type I, characterized by both facial and leptomeningeal angiomas, may be accompanied by glaucoma; Type II, involving facial angiomas alone, possibly with glaucoma; and Type III, marked by isolated leptomeningeal angiomas and the absence of glaucoma [17].

Our patient fits well in the Type I Roach scale.

It is essential to differentiate SWS from infantile hemangiomas, the most common benign tumors in childhood that usually resolve over time. From a molecular perspective, endothelial cells in infantile hemangiomas express Glut-1 positively, whereas endothelial cells in SWS do not [18]. Other differential diagnoses of SWS include Rendu-Osler-Weber syndrome, Von Hippel-Lindau disease, Maffucci syndrome, angio-osteodystrophy syndrome, and Klippel-Trenaumy-Weber syndrome [1,3,19]. Rendu-Osler-Weber syndrome is characterized by the presence of multiple telangiectasias in mucocutaneous sites such as the face, lips, tongue, palms, and fingers, as well as the gastrointestinal tract (GI), lungs, brain, lungs, and liver, which can bleed easily. The patient usually presents with nose bleed, GI bleed, iron deficiency anemia, or intracranial bleed in severe cases. Patients with Von Hippel-Lindau disease have hemangioblastomas of the retina, spinal cord, and brain; clear cell renal cell carcinoma and renal cysts; pancreatic cysts; endolymphatic sac tumor; pheochromocytoma, and neuroendocrine tumors. In Maffucci's syndrome, the patient presents with fractures/deformities due to enchondromatosis. The cavernous hemangiomas of the subcutis, dermis, or internal organs are also present. Congenital vascular bone syndrome or angio-osteodystrophy syndrome is characterized by increased or decreased bone growth due to arterio-venous malformations in long bones. Klippel Trenaumy-Weber syndrome is a triad of bony and soft tissue hypertrophy, mostly atypical lateral varicosity, and capillary malformation (PWS); however, no CNS affection is seen [19].

The presence of a PWS on the forehead raises suspicion of SWS [6]. Employing imaging studies proves helpful in confirming the diagnosis of SWS and assessing the extent of intracranial involvement, particularly when clinical signs are atypical or not fully evident [20].

On skull radiographs, the presence of gyriform calcifications is often referred to as the "tram-track sign" or "railroad-track sign". CT is the preferred imaging technique for detecting calcifications and demonstrating additional alterations like cortical atrophy and leptomeningeal enhancement in post-contrast studies [21]. Currently, the preferred neuroimaging approach for diagnosing SWS is employing MRI with gadolinium contrast, as it can demonstrate the presence of leptomeningeal angiomatosis and determine the degree of involvement of brain structures [6]. The characteristic electroencephalogram (EEG) of patients with SWS is asymmetric, with decreased voltages and focal discharges in the affected brain hemisphere. EEG is also very useful for distinguishing migraines and seizures after cerebrovascular events as causes of acute paroxysmal events [15]. Functional neuroimaging studies of glucose metabolism by positron emission tomography (PET) or cerebral perfusion by (single photon emission computed tomography), show transient hypermetabolism (PET) in the affected cortex secondary to hyperperfusion. At advanced stages, there is hypometabolism and hypoperfusion [13,21].

Radiographic imaging depicting the characteristic gyriform calcifications confirmed the diagnosis of SWS in the present case.

There is no defined treatment protocol for SWS, and the primary goal is to reduce seizure activity with anticonvulsant therapy. Seizure control is achieved in up to 50% of patients using medications, and with the use of newer anticonvulsants and low-dose aspirin, this percentage is likely higher. However, when medical management proves ineffective and refractory seizures persist, surgery becomes a viable option [21].

The presence of a PWS can inflict profound psychological distress on the patient, and it impacts the personality development of nearly all individuals. Dermabrasion, tattooing, and flash lamp-pulsed dye lasers for photocoagulation are the possible approaches that may improve PWS appearance [3].

Various approaches employed for treating gingival hyperplasia include vigilant observation, steroids, radiation therapy, antimetabolites, injection of sclerosing solutions (sodium tetradecyl sulfate) into the gingival tissue, and surgical interventions including LASERS (neodymium-doped yttrium aluminum garnet (Nd:YAG) laser, CO2 laser), cryosurgery (nitrous oxide gas at a temperature of -89°C at the probe tip), or electrosurgery [14,22].

In the present case, a comprehensive plaque control program (regular deep scaling and root planing, patient motivation to maintain optimal oral hygiene, the use of 0.2% chlorhexidine mouth rinse after brushing), along with a balanced diet regimen was followed to treat gingival hypertrophy.

Restoring the dental health of patients is a complex process, and necessitates an initial conservative approach, followed by surgical intervention [22]. Dental management in SWS may be associated with a potential risk of life-threatening intra- and postoperative hemorrhage, making it imperative to implement preventive measures and thoroughly grasp potential outcomes before undertaking any dental procedure.

The various methods can be used to manage the risk of hemorrhage like patient’s blood typed and cross-matched, provision for blood transfusion, use of hemostatic agents (tranexamic acid), injecting sclerosing solutions, percutaneous transcatheter vascular embolization using gel-foam or polyvinyl alcohol [1].

Patient education and motivation to maintain meticulous oral hygiene and plaque control measures may be sufficient for mild-moderate medium lesions; however, the recommended approach for massive gingival enlargements involves a combination of gingivectomy and laser therapy [3].

Recommendations

SWS is the third most common neurocutaneous disorder. Unilateral PWS along the ophthalmic dermatome of the trigeminal nerve should raise a suspicion of SWS. Dental management poses a potential risk for bleeding, and preventive measures should be taken to avert complications.

## Conclusions

The prevalence of oral manifestations in SWS highlights the importance of dentists having a thorough understanding of the clinical features and treatment options for this condition. Effective treatment necessitates consultations with various medical specialists, coupled with professional counseling, to aid both the patient and the family in overcoming difficulties and enhancing the prognosis. Regular systemic and oral examinations are advised to mitigate the risk of cranial and oral complications.
